# Diaphragm electromyography guidance for a lung transplant recipient with difficult weaning from mechanical ventilation: A case report

**DOI:** 10.1097/MD.0000000000010989

**Published:** 2018-06-18

**Authors:** Yuanda Xu, Qi Qing, Minyong Liang, Weibo Liang, Zhimin Lin, Weiliang Wu, Weiqun He, Xiaoqing Liu, Yuanming Luo, Yimin Li, Jianxing He

**Affiliations:** a Department of Critical Care Medicine, First Affiliated Hospital of Guangzhou Medical University, Guangzhou; b Department of Respiratory and Critical Care Medicine, People's Hospital of Xiangxi Tujia Autonomous Prefecture, Jishou; c State Key Laboratory of Respiratory Disease, Guangzhou Institute of Respiratory Disease; d Department of Thoracic Surgery, The First Affiliated Hospital of Guangzhou Medical University, Guangzhou, China.

**Keywords:** diaphragm electromyography, end-stage lung disease, lung transplantation, weaning

## Abstract

**Rationale:**

Many factors contribute to a complicated postoperative course following difficult weaning off a ventilator after lung transplantation.

**Patient concerns:**

A female patient underwent a successful surgery but received a size-mismatched lung graft. The graft had been pruned before transplantation. She experienced delayed ventilator weaning 3 days after lung transplantation.

**Diagnoses:**

A postoperative X-ray revealed a normal mediastinal structure and diaphragm position. Diaphragmatic function was assessed by diaphragm electromyography (EMGdi) via esophageal and surface electrodes. EMGdi showed decreased left compound motor action potentials (CMAPs), prolonged left phrenic nerve conduction time (PNCT), failure to induce right CMAPs and PNCT under bilateral magnetic stimulation, and right phrenic nerve injury.

**Interventions:**

She was treated with neural nutritional support and prescribed rehabilitation measures such as strengthening limb activities on the bed.

**Outcomes:**

The patient finally achieved satisfactory outcomes after an early diagnosis and medical interventions.

**Lessons:**

Lung size mismatch before transplantation and phrenic nerve injury during surgery should be avoided wherever possible.

## 1 Introduction

Lung transplantation is one of most effective therapies for patients with end-stage lung disease.^[1]^ Many factors contribute to a complicated postoperative course after difficultly in weaning a patient off a ventilator, such as development of severe infection, application of intraoperative cardiopulmonary bypass, basic disease status and medication, severe malnutrition, use of immunosuppressive agents, high dose of corticosteroids, graft dysfunction, graft size mismatching, and intensive care unit (ICU)-acquired myasthenia. These factors may result in prolonged postoperative ventilator use, increased comorbidities, and unfavorable prognoses. Thus, efforts should be made to identify risk factors that predict delayed ventilator weaning after lung transplantation.

In this study, our patient underwent uneventful surgery, but experienced delayed ventilator weaning 3 days after lung transplantation. The diaphragmatic dysfunction was assessed by diaphragm electromyography (EMGdi) via esophageal and surface electrodes under magnetic stimulation of the phrenic nerves. The patient finally achieved satisfactory outcomes after an early diagnosis and subsequent interventions.

## 2 Case presentation

This study was approved by the Scientific Research Project Review Ethics Committee of the First Affiliated Hospital of Guangzhou Medical University (ethics batch number 2017 No. 34). Written consent was obtained.

### 2.1 At admission

A 44-year-old female patient (weight 47 kg, height 158 cm) was admitted to our hospital in September 2016 complaining mostly of repeated cough, sputum, and shortness of breath during the previous 5 years. She had an acute exacerbation of these symptoms as recently as 10 months ago. Five years ago, she started coughing up small amounts of white phlegm for no obvious reason and had recurrent and progressive shortness of breath after walking (less than 30 m) and physical activities. In May 2013, the chest computed tomography (CT) showed bilateral interstitial fibrosis in the lungs. The patient was treated with prednisolone and cyclophosphamide, but failed to achieve an optimal therapeutic response. Another chest CT in July 2015 indicated that her interstitial fibrosis had worsened. The patient developed aggravating dyspnea and shortness of breath 10 months before admission, which required continuous oxygen therapy. The patient was finally diagnosed with end-stage, connective tissue disease-associated interstitial lung disease (systemic sclerosis or Sjögren syndrome suspected).

### 2.2 Transplantation

After routine preoperative assessment for lung transplantation, the patient underwent a right side, allogeneic lung transplantation on September 20, 2016. The lung donor was a male aged 21 years old and with a height of 172 cm. The donor died due to a severe craniocerebral injury, and the graft was well-persevered following donation after brain death (DBD). The lung transplantation procedure was uneventful, and the total operation time was 5.25 hours.

The patient exhibited good postoperative diastolic function with the grafted lung. The patient was transferred into ICU with a tracheal cannula, and was given ventilator-assisted breathing with the following parameters: mode, intermittent positive-pressure ventilation (IPPV); tidal volume (TV), 320 mL; respiratory rate (RR), 20/minute; positive-end expiratory pressure (PEEP), 6 cm H_2_O. She was treated against acute rejection and preventive antiinfection therapy. The patient breathed smoothly, with the right main bronchial anastomosis in good condition according to a bronchoscopy.

### 2.3 Treatment in ICU

At the 4th day posttransplantation, the patient exhibited a stable cardiovascular circulation, and sedative analgesia was discontinued. The ventilation mode was adjusted to pressure support ventilation (PSV) 12 cm H_2_O, with PEEP at 5 cm H_2_O, PaO_2_/FiO_2_ ratio of 280 mm Hg, negative inspiratory force (NIF) 13 cm H_2_O, and a normal partial pressure of carbon dioxide (PaCO_2_) in arterial blood. She was then given bilevel positive airway pressure (BiPAP) ventilation by noninvasive nasal BiPAP mask.

However, the patient suffered the reestablishment of mechanical ventilation via an artificial airway 6 hours later due to hypoxemia and hypercapnia. A bedside chest X-ray showed a normal mediastinal structure, without increase in exudation of both lungs. The position of the right hemidiaphragm was normal. The echocardiography revealed normal cardiac function.

Considering possible diaphragmatic dysfunction, EMGdi was recorded from both esophageal and surface electrodes. The twitch transdiaphragmatic pressure (TwPdi) measurements under magnetic stimulation of the phrenic nerves showed a bilateral phrenic nerve conduction time (PNCT) of 13 milliseconds and bilateral diaphragmatic compound motor action potentials (CMAPs) of 0.508 mV (Table 1). After adjustment of the PSV of the ventilator from 12 cm H_2_O to 10 cm H_2_O, the diaphragm electromyogram (EMGdi) value was elevated from 9.8 ± 0.71 uv to14.6 ± 1.22 uv, achieving a statistical significance (*P* < .05), and the rapid shallow breathing index (RSBI) was increased from 78.1 ± 6.05 to 120.7 ± 14.70 (*P* < .05).

**Table 1:**
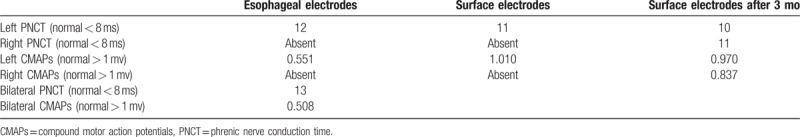
The unilateral and bilateral PNCT and CMAPs recorded by diaphragm electromyography.

With further adjustment of the PSV of the ventilator to 8 cm H_2_O for 30 minutes, the EMGdi value continued to elevate from 14.6 ± 1.22 uv to 16.9 ± 1.06 uv (*P* < .05); and the RSBI increased from 120.7 ± 14.70 to 146.7 ± 14.00 (*P* < .05). Two hours later, the EMGdi value increased from 16.9 ± 1.06 uv to 17.1 ± 1.28 uv, without statistical significance (*P* = .548); RSBI fluctuated in a small amplitude from 146.7 ± 14.00 to 134.8 ± 8.88, *P* = .370 (Table 2).

**Table 2:**
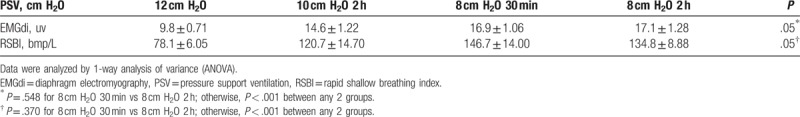
EMGdi and RSBI under different levels of pressure support ventilation.

The patient had normal muscle strength according to the Medical Research Council (MRC) scale of 60 points, and without the presence of ICU-acquired weakness symptoms. She was treated with neural nutritional support and prescribed rehabilitation measures such as strengthening limb activities on the bed.

Fourteen days later, the patient successfully completed a spontaneous breathing trial for 2 hours, twice per day. The trachea cannula was removed, and sequentially noninvasive BiPAP ventilation was given. The patient had no signs of shortness of breath, chest tightness, or palpitation. Three months later, the patient walked to the hospital unaided for further examination. The EMGdi recorded from surface electrodes showed a maximal inspiratory pressure (MIP) of −20.0 cm H_2_O, left PNCT of 10.0 milliseconds, left CMAP of 0.970 mV, right PNCT of 11.0 milliseconds, left CMAP of 0.837 mV (Table 1). Thus a significant improvement in her right diaphragmatic paralysis had occurred.

## 3 Discussion

This case is a patient who failed to wean from mechanical ventilation 3 days after lung transplantation, and then suffered the reestablishment of mechanical ventilation via an artificial airway. The phrenic nerve injury following lung transplantation may result in prolonged postoperative mechanical ventilation and weaning difficulty.^[2]^ This patient had a low PSV of 12 cm H_2_O, and a reduction of 2 cm H_2_O in PSV, monitored by diaphragmatic EMG, resulted in significant increase in EMGdi value and RSBI revealing insufficient respiratory muscle strength and declined TV. The signals were sent to the respiratory center to produce a stronger feedback electrical signal. The EMGdi showed a decreased left CMAPs and prolonged left PNCT and failure to induce right CMAPs and PNCT under bilateral magnetic stimulation, revealing right phrenic nerve injury.

Damage to the phrenic nerve, either unilaterally or bilaterally, is a well-documented complication of cardiac operations, largely because the nerve is close to the pericardium. However, phrenic nerve injury is less commonly reported after lung transplantation. It has been reported that among retrospective data of 49 lung transplantations, phrenic nerve paralysis was found in 4 (8.2%) patients (unilateral in 3 patients and bilateral in 1).^[3]^ Pulmonary and other factors that should be considered in difficult-to-wean patients following lung transplantation include unstable cardiac function, sepsis, anastomotic dehiscence, airway secretions, hemothorax or pneumothorax, and mediastinal swing.^[4,5]^

In addition to these factors, physicians should be aware of the possibility of phrenic nerve injury resulting from intraoperative mechanical stretching, freezing, and prolonged chest surgery. Marco et al^[6]^ showed that the canine phrenic nerve undergoes microscopic nerve myelin changes when exposed to a frozen environment for 30 to 60 minutes. Phrenic nerve injury may be closely associated with the basic disease and medication history of patients.^[7]^ Accumulating evidence has showed that unilateral diaphragmatic dysfunction exists in 50% patients without overt clinical manifestations and 25% patients with mild exertional dyspnea, and is probably likely to result in dyspnea in patients with severe respiratory diseases.^[8–10]^

In our case it should be noted that the size of donor lung did not match the size of the recipient's diseased lung, as the height of the donor was 172 cm whereas the recipient's height was 158 cm. In addition, the chest cavity of the recipient was (too) small to accommodate the lung of the donor. Moreover, the patient had end-stage, connective tissue disease-associated interstitial lung disease (systemic sclerosis or Sjögren syndrome suspected), which made the chest even smaller. However the graft had been pruned before transplantation, and a postoperative X-ray indicated a normal mediastinal structure and diaphragm position, without mediastinal shift or raised or collapsed diaphragm on the right side. There were also no signs of abnormal breathing under positive pressure ventilation.

On the other hand, implanting an excessively sized lung carries a high risk of prolonged mechanical ventilation.^[11]^ Thus, while it is reasonable for patients with end-stage chronic obstructive pulmonary disease (COPD) to receive a larger lung graft, a relatively smaller lung graft is more appropriate for patients with advanced pulmonary fibrosis.^[12]^

## 4 Conclusion

For patients with delayed weaning from mechanical ventilation following lung transplantation, EMGdi under magnetic stimulation can be applied to accurately evaluate phrenic nerve and diaphragm function. Efforts should be made to achieve early diagnoses and interventions in these difficult-to-wean patients. A lung size mismatch before transplantation and phrenic nerve injury during surgery should be avoided wherever possible.

## Author contributions

**Conceptualization:** Yuanda Xu, Qi Qing.

**Data curation:** Yuanda Xu, Qi Qing, Minyong Liang, Zhimin Lin, Weiliang Wu.

**Formal analysis:** Weiqun He, Xiaoqing Liu, Yuanming Luo.

**Funding acquisition:** Yimin Li, Jianxing He.

**Investigation:** Weibo Liang, Zhimin Lin.

**Methodology:** Yuanda Xu, Qi Qing, Minyong Liang.

**Project administration:** Yimin Li,Jianxing He.

**Resources:** Yuanming Luo, Yimin Li, Jianxing He.

**Software:** Yuanda Xu, Qi Qing, Minyong Liang.

**Supervision:** Yuanda Xu, Qi Qing, Yimin Li, Jianxing He.

**Validation:** Yuanda Xu, Qi Qing.

**Visualization:** Yuanda Xu, Qi Qing.

**Writing – original draft:** Yuanda Xu, Qi Qing.

**Writing – review & editing:** Yuanda Xu, Qi Qing.
